# Parancentesis in Internal Hydrocephalus

**Published:** 1889-04

**Authors:** Samuel Ayres

**Affiliations:** Pittsburg, Pa.


### PARACENTESIS IN INTERNAL HYDROCEPHALUS.*

BY SAMUEL AYRES, M. D., PITTSBURG, PA.

GENTLEMEN: With your permission I will report this evening a case of acquired chronic internal hydrocephalus, for the relief of which I undertook paracentesis, after trephining. My excuse for presenting it to you is that the operation is quite rare, and a considerable fatality has attended it, though in this case we have the satisfaction of observing a decided improvement. The history of the boy, who is present this evening, is as follows: His age is nearly five years, his height 3 feet 1½ inches, and weight 43 pounds. There seems to be no history of the neuroses in his family. His father's mother died of cancer of the breast, and an aunt of his mother, of phthisis. His mother and father have always enjoyed the best of health, the latter being a laborer. He denies any venereal infection.

*A paper read at Allegheny County Medical Society, Feb. 19 1889.

The mother states that her boy was perfectly healthy and normally developed up to three months of age, when, without known cause, he was suddenly seized with convulsions during sleep. These attacks became very frequent, occasionally from twenty to thirty occurring daily, and they continued for nine months, or until he was one year old. They then ceased, and have not recurred. Three months after their commencement his head had naturally enlarged and assumed a pear shape, the greater diameters corresponding with the parieto-occipital planes.

There was no separation of the cranial bones. From the date of the beginning of his trouble up to the time that he was brought to my clinic, in October, 1888, every form of treatment had been faithfully tried, according to his parents, but without the least success. At that time his condition was this: Mentally, there had been little or no development. He was obviously imbecile. He could not talk, but smiled idiotically. He was totally blind, but the other special senses were not apparently affected. He had never walked or stood alone, but could easily move his body and extremities. The bowel and bladder sphincters were not controlled. He was extremely irritable and restless, and slept very irregularly. He would take only liquid food given from a spoon. He was fairly developed physically and quite well nourished, but always of an ashy pallor. There was a very frequent rotary movement of the head, with slight retraction, and grinding of the teeth. His pulse was from 120 to 140 per minute. Temperature was not taken. The measurements of the head were the following: From the glabella to inion, 12½ inches; over the biauricular line, 13¾ inches; around the fronto-occipital line, 20 inches. The anterior fontanelle closed when he was eighteen months old, and the sutures had ossified at the usual time. No abnormal sensory symptoms were noticed. We could not succeed in making an examination of the eyes owing to his restless state.

A diagnosis of ventricular effusion was ventured. His parents suggested and urged some kind of an operation as a *dernier resort*, as they fancied something might be done surgically. To this I very reluctantly consented, having distinctly pointed out the dangers and uncertainties incident to such procedure.

On the 4th of December, 1888, assisted by Drs. Murdoch, Hersman and Boreland, and in the presence of the students of the Western Pennsylvania Medical College, the following course was pursued: The head having been shaved, washed with strong soap, and enveloped in carbolized cotton for twenty-four hours, the boy was chloroformed. Over the coronal suture, about one and one-half inches to the right of the median line, a small flap of the scalp and pericranium was reflected. With a trephine about one cm. in diameter, I removed a button of bone, which was slightly thicker than normal. The dura looked healthy, but owing to its small exposure the tension could not well be estimated. It distinctly pulsated. A very delicate trocar was passed through the dural membrane into the brain substance, downwards, backwards and inwards, to the depth of two and one-half inches, the object being to pierce the central cavity of the right lateral ventricle.

There were no reflex movements of any kind observed during this procedure. The trocar being removed, a clear, limpid fluid began to ooze, drop by drop, from the canula. About an ounce of this was evacuated, when the canula was slowly withdrawn. As its internal orifice reached the sub-dural space, the same transparent fluid dropped from the opposite end, thus revealing the presence of perhaps an excess of fluid in this space also.

Its specific gravity was not obtained, but it was faintly alkaline, non-albuminous, contained chlorides in abundance, and also phosphates. Evidently it closely resembled the cerebro-spinal fluid.

The pericranium and scalp were approximated and stitched, and the wound dressed with carbolized gauze. For several days the same fluid continued to ooze from the puncture in the dura, and to saturate the dressings. It was estimated that from four to eight ounces was thus discharged.

The case progressed satisfactorily, the pulse not exceeding 140, nor the temperature rising above $101\frac{8}{10}°$. The child seemed anxious to be up in two or three days, and could then stand alone. The results of the operation were quickly observed in a partial restoration of sight. The boy could evidently see, as he would wink when a finger was passed before his eyes. Then, having stood unassisted, he was gradually able to walk alone across the room, which he did in about three weeks. He became less irritable, and instead of short snatches of sleep, as before, slept the entire night. He also took some solid food, which he had not done previously. For three weeks following the tapping he did not rotate the head a single time.

The mother states that, in a great many respects, the child is improved. He is more attentive, and seems to better understand her. There is, however, no development of speech, nor are the sphincters under any better control.

He continued in this improved state till the latter part of January last, when, after some slight ailment, he gradually lost the power to walk. Thinking that the fluid was re-accumulating, we proposed tapping him again on the eighth of February, but on the fifth he commenced to walk again, so it was postponed.

It is my opinion that more fluid will have to be evacuated, as he is not quite so active now as some time after the tapping. I am fortunate in being able to report an ophthalmoscopic examination of his eyes, which was very kindly made to-day by Dr. Lippincott, while the boy was anæsthetized for the purpose.

"Right eye. Disc snow white, and with sharply defined margins. Capillary circulation greatly diminished. Retinal vessels, especially veins, reduced in calibre. Arteries not very much below normal size. Retina also atrophic. Media clear.

"Left eye. Same condition as right, except that the disc margins are even more sharply defined and a small quantity of pigment is seen surrounding the disc.

"Divergent strabismus of O. S. Pupils normally react."

The most practical bearing this operation would seem to have is in reference to those imbecile children, the commencement of whose trouble dates back to some acute or sub-acute cerebral affection, as simple meningitis or convulsions, or perhaps slight traumatism, but whose cases have always been considered irremediable. It seems probable that many such cases go to fill the institutions for feeble-minded throughout our land, or else prove, in their own homes, a grave care and an eternal responsibility to parents. And the question may be pertinently asked whether an excessive or pathological effusion within the cerebral ventricles or sub-dural space, or within both, may not only have caused, but perpetuated, these undeveloped brains and minds.

The presence of such excess of fluid is abundantly adequate to produce the symptoms by pressure upon the delicate nerve elements or tracts, and the varying character of the attacks, as in this case, would strengthen the probability that a fluid, changing with the body position or blood pressure, was the responsible agent in their production. Or, during the sub-acute stage, when convulsions, coma, paralysis, or other grave symptoms follow an obscure brain attack, consisting, perhaps, in ventricular inflammation, and when death is imminent, it seems to me not only justifiable but imperative, that the sub-dural space be tapped, and if the symptoms do not soon abate, that the lateral ventricles be penetrated. If the product of such inflammation should prove to be purulent, which is not probable, the opening in either case would have to be enlarged and thorough drainage, with antiseptic irrigation, established.

But since most of these sub-acute cases of sub-dural or ventricular meningitis are attended only with fluid effusion, the operation would be comparatively easy, particularly if the fontanelles had not ossified. I shall never again stand by, even after exhausting all other resources, and allow convulsions or coma, or paralyzed respiratory centers to carry off an infant, with all the symptoms of ventricular effusion, without resorting to paracentesis, unless a tuberculous history makes certain his fate.

In the performance of the operation great care should be exercised. The chief difficulty lies in our inability to determine which cavity to evacuate. For instance, if the fluid resides in both cavities, and the normal openings between them, through the foramen of Majendie, and those behind the roots of the glosso-pharyngeal nerves be closed by inflammatory exudation, or the presence of a tumor, then to tap only the subdural space would remove the external pressure, and allow such an expansion of the internal fluid as would perhaps lacerate the brain tissue. Or the same effect might be produced by evacuating only the ventricular fluid. This may have been the cause of death in some of the reported cases.

It would seem, therefore, that the safest course to pursue would be that followed in the above, particularly when the cranial sutures are closed—to tap first the ventricles and then the external space.

The operation for tapping for hydrocephalus is not new. It is thought Le Cat first performed it in 1744. West (*3d Am. Ed.*, p. 117) refers to the operation, but states that opinion is divided as to the propriety of the practice. He had up to that time collected 56 reported cases from different sources, with 4 recoverеries, but he does not state whether they are of the external or internal form; though the inference is that they were of the former, since compression after the operation is referred to.

Watson (p. 291 *et seq.*) gives several cases of the external form, for which tapping was practiced, with variable results, some dying, some recovering, but he speaks of no cures.

Ellis (3d Ed., p. 83) says that puncturing is not applicable to the ventricular form, and he rather discourages its employment in the external. He quotes Churchill, who gives a list of unsuccessful cases. .But Ellis admits that the operation has been occasionally successful.

In the Cyclopædia of Practical Medicine (vol.II, p. 500) Folis is quoted as giving the names of 27 writers who had expressed themselves in favor of the operation. Yet he himself, with Boerhaave, Dupuytren, Heister, Hecker and Portenshlag, regarded it as cruel and useless.

Geo. B. Wood (2d Ed., vol. II, p. 343) says that tapping has been employed by many with uncertain results.

Elliotson (2d Ed., p. 511) states that he never saw a case of the kind, but that if a minute puncture be made and a small quantity of fluid be evacuated at a time, it might be done with safety and with prospects of relief.

Reynolds (*System of Med.*, vol. 1, p. 840) quotes Watson, West and Conquest on the subject, the last of whom he states has been the greatest advocate of the operation in this country (England).

Condie 2d Ed., (p. 400) refers directly to the tapping of the ventricles and points out with remarkable accuracy the procedure. At the same time he admits that he had never seen a case.

Trosseau (*Clinical Med.*, vol. 1, p. 890) remarks that the brain has been tapped through the sutures and fontanelles for hydrocephalic accumulation, but he does not seem to think its advantages counterbalance its disadvantages.

Henoch (*Handbook for Phys. & Student*, p. 116, Wm. Wood & Co., 1882) says of chronic internal hydrocephalus: "At least I have achieved no results whatever, either with mercurial inunction, iodide of potassium or applications of tincture of iodine to the head. Nor can I promise any results from compression of the skull with strips of adhesive plaster, or *from puncture through the fontanelles.*"

Gross (5th Ed., vol. II, p. 123) has practiced paracentesis in two cases, both dying within two days after the operation.

Agnew (vol. II, p. 886) speaks of the benefits of the operation as claimed by West and Conquest, but declares such results have never been obtained in this country. He describes only the tapping of external hydrocephalus when the sutures or fontanelles are not closed.

Meigs and Pepper (7th Ed., 1882, p. 557) refer to West's cases and give one of internal hydrocephalus, upon which they operated, death following in less than forty-eight hours. Examination, however, proved that the case was hopeless, the brain being disorganized at the base and the child, less than three years old, the victim of miliary tuberculosis.

J. Lewis Smith (6th Ed., pp. 448 and 449) advises the performance of the operation, and regards it as simple and devoid of danger, but his remarks apply only to the congenital form, and evidently to those of opened sutures and fontanelles. He makes no record to tapping in acquired hydrocephalus.

Gowers (*A Manual of Diseases of the Nervous System*, vol. II, 1888, p. 544) considers evacuation by puncture the most direct, but unfortunately the most dangerous, treatment, and advises that but a small quantity be let out each time, and compression of the skull by elastic bandages be kept up. This procedure, he says, is of cours most suitable in external effusion, but it has been employed in the ventricular also, occasionally without ill effect, but with absolute success only in rare instances.

An operation in a different kind of case altogether, but with very similar technique, excepting that in this the dura was incised, is referred to by Senn,* an imperfect account of which is obtained from the *Melbourne Age*, a non-professional Australian journal.

This operation was done for an echinococcus cyst in the brain on January 27, 1888. The patient, a girl of sixteen, was chloroformed. A button of bone one inch in diameter was removed from the left temple, the dura incised, and a trocar inserted through the substance of the brain, and the cyst successfully penetrated, a large quantity of fluid coming away.

Thus it will be seen that a diversity of opinion is expressed by authors as to the propriety of paracentesis capitis. But in the light of modern antisepsis, and of a more intimate knowledge of the brain anatomically and physiologically, it would seem that this operation must take its legitimate place among those which were once conceived to be both impracticable and impossible.

I can find reference to no other case similar to the one above reported, in which tapping was practiced for both ventricular and sub-dural effusion of the primary or acquired character, the sutures and fontanelles being closed, and trephining being required to permit of the entrance of the trocar.

*Annual of the Universal Medical Sciences, 1888, Vol. II, page 36.

Now is the time to subscribe for the JOURNAL.

